# Supplementary material for: Decomposition of the mean absolute error (MAE) into systematic and unsystematic components
Source: PLoS One. 2023 Feb 17;18(2):e0279774. doi: 10.1371/journal.pone.0279774 (PMC9937461; doi:10.1371/journal.pone.0279774)
Supplement: S1 File — (PDF) [file pone.0279774.s001.pdf]

Supporting Information for

Decomposition of the mean absolute error (MAE) into systematic and unsystematic components

Scott M. Robeson[1*], Cort J. Willmott[2]
[1] Department of Geography, Indiana University, Bloomington, Indiana, USA
[2] Department of Geography, University of Delaware, Newark, Delaware, USA
* srobeson@indiana.edu

Below are R and Matlab functions for the decomposition of mean absolute error (MAE) into three components: bias error (MAE_b), proportional error (MAE_p), and unsystematic error (MAE_u).

```
# R function to decompose MAE into three components
# Robeson and Willmott, PLOS ONE
#
# Inputs are the observations (obs) and the model predictions (pre)

mae_decomp <- function(obs, pre) {

# setup
n <- length(obs)
abserr <- abs(pre - obs)
mbe <- mean(pre) - mean(obs)
pre_prime <- pre - mbe

# OLS regression to get phat_prime
reg <- lm(pre_prime ~ obs)
phat_prime <- reg$coefficients[1] + reg$coefficients[2] * obs

# Form weights
b <- abs(mbe)
p <- abs(phat_prime - obs)
u <- abs(pre_prime - phat_prime)
denom <- (b + p + u)
bias <- b / denom
prop <- p / denom
unsys <- u / denom

# Components of MAE
maeb <- sum(bias * abserr) / n
maep <- sum(prop * abserr) / n
maeu <- sum(unsys * abserr) / n

# Output
res <- c(maeb, maep, maeu)
names(res) <- c("MAE_b", "MAE_p", "MAE_u")
res
}
```

```
function [ maeb, maep, maeu ] = mae_decomp( obs, pre )
%MAE_DECOMP Matlab function to decompose MAE into
% systematic and unsystematic components
% Robeson and Willmott, PLOS ONE

% setup
n = length(obs);
abserr = abs(pre - obs);
mbe = mean(pre) - mean(obs);
pre_prime = pre - mbe;

% OLS regression to get phat_prime
coeff2 = polyfit(obs,pre_prime,1);
phat_prime = coeff2(2) + coeff2(1) * obs;

% Form weights
b = abs(mbe);
p = abs(phat_prime - obs);
u = abs(pre_prime - phat_prime);
denom = (b + p + u);
bias = b./denom;
prop = p./denom;
unsys = u./denom;

% Components of MAE
maeb = sum(bias.*abserr)/n;
maep = sum(prop.*abserr)/n;
maeu = sum(unsys.*abserr)/n;

end
```
