# Supplementary material for: Decomposition of the mean absolute error (MAE) into systematic and unsystematic components
Source: PLoS One. 2023 Feb 17;18(2):e0279774. doi: 10.1371/journal.pone.0279774 (PMC9937461; doi:10.1371/journal.pone.0279774)

PLOS ONE

Decomposition of the mean absolute error (MAE) into systematic and unsystematic components

--Manuscript Draft--

**Manuscript Number:**	PONE-D-22-17609
**Article Type:**	Research Article
**Full Title:**	Decomposition of the mean absolute error (MAE) into systematic and unsystematic components
**Short Title:**	Decomposition of mean absolute error
**Corresponding Author:**	Scott M. Robeson<br>Indiana University Bloomington<br>Bloomington, Indiana UNITED STATES
**Keywords:**	model error; error decomposition; model respecification
**Abstract:**	When evaluating the performance of climatic and other geophysical models, dimensioned errors often are characterized by sums-of-squares measures such as the mean squared error (MSE) or its square root, the root mean squared error (RMSE). In terms of quantifying average error, however, absolute-value-based measures such as the mean absolute error (MAE) are more interpretable than MSE or RMSE. Part of that historical preference for sums-of-squares measures is that they are mathematically<br><br>amenable to decomposition and one can then form ratios, such as those based on separating MSE into its systematic and unsystematic components. Here, we develop and illustrate a decomposition of MAE into three useful submeasures: (1) bias error, (2) proportionality error, and (3) unsystematic error. This three-part decomposition of MAE is preferable to comparable decompositions of RMSE because it provides more straightforward information on the nature of the model-error distribution. We illustrate<br><br>the properties of our new three-part decomposition using a long-term reconstruction of streamflow for the Upper Colorado River.
**Order of Authors:**	Scott M. Robeson
	Cort J. Willmott
**Additional Information:**	
**Question**	**Response**
**Financial Disclosure**<br><br>Enter a financial disclosure statement that describes the sources of funding for the work included in this submission. Review the submission guidelines for detailed requirements. View published research articles from *PLOS ONE* for specific examples.<br><br>This statement is required for submission and **will appear in the published article** if the submission is accepted. Please make sure it is accurate.	The author(s) received no specific funding for this work.

Powered by Editorial Manager® and ProduXion Manager® from Aries Systems Corporation

**Unfunded studies**<br>Enter: *The author(s) received no specific funding for this work.*<br><br>**Funded studies**<br>Enter a statement with the following details:<br>• Initials of the authors who received each award<br>• Grant numbers awarded to each author<br>• The full name of each funder<br>• URL of each funder website<br>• Did the sponsors or funders play any role in the study design, data collection and analysis, decision to publish, or preparation of the manuscript?<br>• **NO** - Include this sentence at the end of your statement: *The funders had no role in study design, data collection and analysis, decision to publish, or preparation of the manuscript.*<br>• **YES** - Specify the role(s) played.<br><br>* typeset	
**Competing Interests**<br><br>Use the instructions below to enter a competing interest statement for this submission. On behalf of all authors, disclose any competing interests that could be perceived to bias this work—acknowledging all financial support and any other relevant financial or non-financial competing interests.<br><br>This statement is required for submission and **will appear in the published article** if the submission is accepted. Please make sure it is accurate and that any funding sources listed in your Funding Information later in the submission form are also declared in your Financial Disclosure statement.<br><br>View published research articles from *PLOS ONE* for specific examples.	The authors have declared that no competing interests exist.

**NO authors have competing interests**<br><br>Enter: *The authors have declared that no competing interests exist.*<br><br>**Authors with competing interests**<br><br>Enter competing interest details beginning with this statement:<br><br>*I have read the journal's policy and the authors of this manuscript have the following competing interests: [insert competing interests here]*<br><br>* typeset	
**Ethics Statement**<br><br>Enter an ethics statement for this submission. This statement is required if the study involved:<br><br>• Human participants<br>• Human specimens or tissue<br>• Vertebrate animals or cephalopods<br>• Vertebrate embryos or tissues<br>• Field research<br><br>Write "N/A" if the submission does not require an ethics statement.<br><br>General guidance is provided below. Consult the submission guidelines for detailed instructions. **Make sure that all information entered here is included in the Methods section of the manuscript.**	N/A

Powered by Editorial Manager® and ProduXion Manager® from Aries Systems Corporation

**Format for specific study types**<br><br>**Human Subject Research (involving human participants and/or tissue)**<br>• Give the name of the institutional review board or ethics committee that approved the study<br>• Include the approval number and/or a statement indicating approval of this research<br>• Indicate the form of consent obtained (written/oral) or the reason that consent was not obtained (e.g. the data were analyzed anonymously)<br><br>**Animal Research (involving vertebrate animals, embryos or tissues)**<br>• Provide the name of the Institutional Animal Care and Use Committee (IACUC) or other relevant ethics board that reviewed the study protocol, and indicate whether they approved this research or granted a formal waiver of ethical approval<br>• Include an approval number if one was obtained<br>• If the study involved *non-human primates*, add *additional details* about animal welfare and steps taken to ameliorate suffering<br>• If anesthesia, euthanasia, or any kind of animal sacrifice is part of the study, include briefly which substances and/or methods were applied<br><br>**Field Research**<br><br>Include the following details if this study involves the collection of plant, animal, or other materials from a natural setting:<br>• Field permit number<br>• Name of the institution or relevant body that granted permission	
**Data Availability**<br><br>Authors are required to make all data underlying the findings described fully available, without restriction, and from the time of publication. PLOS allows rare exceptions to address legal and ethical concerns. See the PLOS Data Policy and FAQ for detailed information.	Yes - all data are fully available without restriction

A Data Availability Statement describing where the data can be found is required at submission. Your answers to this question constitute the Data Availability Statement and **will be published in the article**, if accepted.<br><br>**Important:** Stating 'data available on request from the author' is not sufficient. If your data are only available upon request, select 'No' for the first question and explain your exceptional situation in the text box.<br><br>Do the authors confirm that all data underlying the findings described in their manuscript are fully available without restriction?	
**Describe where the data may be found in full sentences. If you are copying our sample text, replace any instances of XXX with the appropriate details.**<br><br>• If the data are **held or will be held in a public repository**, include URLs, accession numbers or DOIs. If this information will only be available after acceptance, indicate this by ticking the box below. For example: *All XXX files are available from the XXX database (accession number(s) XXX, XXX.)*.<br>• If the data are all contained **within the manuscript and/or Supporting Information files**, enter the following: *All relevant data are within the manuscript and its Supporting Information files.*<br>• If neither of these applies but you are able to provide **details of access elsewhere**, with or without limitations, please do so. For example:<br><br>*Data cannot be shared publicly because of [XXX]. Data are available from the XXX Institutional Data Access / Ethics Committee (contact via XXX) for researchers who meet the criteria for access to confidential data.*<br><br>*The data underlying the results presented in the study are available from (include the name of the third party*	The data for the original reconstruction as well as the bias-corrected version developed here are available from NOAA's Paleoclimatology Data site (https://www.ncdc.noaa.gov/dataaccess/paleoclimatology-data).

Powered by Editorial Manager® and ProduXion Manager® from Aries Systems Corporation

and contact information or URL).<br>• This text is appropriate if the data are owned by a third party and authors do not have permission to share the data.<br><br>* typeset	
Additional data availability information:	Tick here if your circumstances are not covered by the questions above and you need the journal's help to make your data available.

Powered by Editorial Manager® and ProduXion Manager® from Aries Systems Corporation

Manuscript

Click here to access/download;Manuscript;Robeson_Willmott_MAE_PLOSON

Decomposition of the mean absolute error (MAE) into systematic and unsystematic components

Scott M. Robeson[1*], Cort J. Willmott[2],

1 Department of Geography, Indiana University, Bloomington, Indiana, USA
2 Department of Geography, University of Delaware, Newark, Delaware, USA

* srobeson@indiana.edu

Abstract

When evaluating the performance of climatic and other geophysical models, dimensioned errors often are characterized by sums-of-squares measures such as the mean squared error (MSE) or its square root, the root mean squared error (RMSE). In terms of quantifying average error, however, absolute-value-based measures such as the mean absolute error (MAE) are more interpretable than MSE or RMSE. Part of that historical preference for sums-of-squares measures is that they are mathematically amenable to decomposition and one can then form ratios, such as those based on separating MSE into its systematic and unsystematic components. Here, we develop and illustrate a decomposition of MAE into three useful submeasures: (1) bias error, (2) proportionality error, and (3) unsystematic error. This three-part decomposition of MAE is preferable to comparable decompositions of RMSE because it provides more straightforward information on the nature of the model-error distribution. We illustrate the properties of our new three-part decomposition using a long-term reconstruction of streamflow for the Upper Colorado River.

Introduction

Across the sciences, model-estimation and -prediction errors are often summarized and analyzed using dimensioned [1] and dimensionless [2] measures. While dimensionless error measures have received considerable attention [3] [4] [5], dimensioned measures are better suited to summarizing the magnitude of model error in meaningful units. When given a set of model predictions ($P_i,\ i = 1, 2, ..., n$), where each P_i corresponds to a reliable observation (O_i), the mean squared error (MSE) and the root mean squared error (RMSE):

$$\mathrm{MSE} = \frac{1}{n}\sum_{i=1}^{n}(P_i - O_i)^2 \tag{1}$$

$$\mathrm{RMSE} = \left[\frac{1}{n}\sum_{i=1}^{n}(P_i - O_i)^2\right]^{\frac{1}{2}} \tag{2}$$

are routinely reported [6]. The mean absolute error (MAE):

$$\mathrm{MAE} = \frac{1}{n}\sum_{i=1}^{n}|P_i - O_i| \tag{3}$$

is reported less often, even though it is a more [illegible]aningful indicator of average error [7].

We and others have shown elsewhere that error statistics based on sums-of-squares have a number of issues that make them less interpretable than those based on absolute values [1] [4] [7] [8] [9] [10] [11]. This is especially the case when they are used as measures of average model error. An additional drawback to using MSE is that its (squared) dimensional units are difficult to interpret. As a result, MAE is the preferred measure of average model error. Even so, sums-of-squares measures continue to be assessed and reported, partially due to inertia, but also to their amenability to mathematical decomposition into additive variance-based measures. In the context of evaluating model error, this property was used by Willmott in 1981 [12] to decompose MSE into systematic (MSE_s) and and unsystematic (MSE_u) components:

$$\text{MSE}_s = \frac{1}{n}\sum_{i=1}^{n}(\hat{P}_i - O_i)^2 \tag{4}$$

$$\text{MSE}_u = \frac{1}{n}\sum_{i=1}^{n}(P_i - \hat{P}_i)^2 \tag{5}$$

such that

$$\text{MSE} = \text{MSE}_s + \text{MSE}_u. \tag{6}$$

For both MSE_s and MSE_u, ordinary least-squares (OLS) regression of the model predictions on the observations typically is used to obtain $\hat{P}_i$ (i.e., a linear esti[illegible]e of P_i on O_i).

As estimated above, MSE_s is typically interpreted as consistent over- and/or under-pred[illegible]on of the observations by the model. The unsystematic component provides an estimate of the model's random error or scatter about the regression line. Forming the ratios of MSE_s/MSE and MSE_u/MSE gives estimates of the fraction of total error (as estimated by MSE) that is identified as systematic or unsystematic. This decomposition has served as a relatively insightful summary of model error (e.g., [13]) and has been used as a guide to model improvement because a model that has a large amount of systematic error usually can be respecified to reduce the consistent over- or under-prediction.

While decomposing MSE into its constituent components has been a useful approach, MSE is a flawed measure of *average* model error. Using MSE to identify systematic and unsystmatic components of error, therefore, can produce misleading summaries of the types of errors that various models contain. Even more importantly, models may be inappropriately adjusted to reduce systematic error that has been misidentified by the MSE-based approach ([illegible] when the impacts of outliers are overemphasized). As a result, our goal here is to develop and present a more rational approach for error decomposition that uses MAE as the baseline for average model error.

Decomposition of MAE into Three Components

Although our goal is to partition MAE into components that represent systematic and unsystematic error patterns, we also want to move beyond the traditional two-part decomposition to further divide systematic errors into two separate components. One can be used to indicate the amount of bias in a model and the other to represent the [illegible]ent to which the model predictions systematically under- or over-estimate observations falling below and above the observed mean. The latter is referred to as proportionality error and is distinct from model bias represented in the under- or overestimation of the observed mean.

For each of these three types of error — bias, proportionality, and unsystematic — we develop a weighting function that can be used to partition MAE into its three components. We offer a diagram (Fig. 1) using a small, synthetic dataset to illustrate the estimation of the three components.

Bias Error

Here, we define bias as the component of systematic error that is contained in the over- or under-prediction of the observed mean. This is often referred to as the mean bias error (MBE):

$$\mathrm{MBE} = \bar{P} - \bar{O} \tag{7}$$

In addition to indicating average over- or under-prediction, MBE can be used to develop a corresponding (to P_i) set of unbiased predicted values:

$$P_i' = P_i - \mathrm{MBE} \tag{8}$$

The magnitude (absolute value) of MBE can additionally serve as the weight that determines the relative importance of bias to the overall MAE:

$$b = |\mathrm{MBE}| \ . \tag{9}$$

The magnitude of bias for our example dataset can be seen in the bottom left panel in Fig. 1, where the model predictions systematically underestimate the mean of the observations by 0.714.

Fig 1. Representations of model-prediction errors showing aspects of the decomposition into systematic and unsystematic components. Predictions (in the upper left panel) can be decomposed in the traditional way using MSE, as shown in the upper-right panel where the lengths of the red and blue dotted vertical lines determine the partitioning of the errors. The bottom-left panel shows the predictions after bias is removed (i.e., P_i'), while the bottom-right panel shows the magnitudes of the bias, proportionality, and unsystematic components of our three-part, weight-based decomposition of MAE.

Proportionality Error

In addition to bias error (caused by an incorrect estimation of the observed mean), there is another systematic error related to consistent under- or over-prediction. This error is reflected in the slope of the regression estimate of P_i' on O_i (note that if the regressions are estimated using OLS, then this slope estimate is the same as that of P_i on O). If the slope of this relationship is anything other than unity, there is *proportionality* error in the model predictions. A slope of less than one indicates that the model systematically overestimates values below $\bar{O}$ and underestimates those above. Conversely, a slope greater than one indicates that the model systematically underestimates values below $\bar{O}$ and overestimates those above. To estimate proportionality error, we use the unbiased predicted regression values:

$$\hat{P}_i' = \hat{P}_i - \mathrm{MBE} \tag{10}$$

Given that the OLS solution for $\hat{P}$ is constrained to pass through $(\bar{O}, \bar{P})$, $\hat{P}'$ passes through $(\bar{O}, \bar{O})$ and, therefore, is unbiased. Weights for the relative importance of proportionality error (for each O_i) are determined using the difference between the unbiased predictions and the observations (the red lines in Fig. 1):

$$p_i = |\hat{P}_i' - O_i| \ . \tag{11}$$

Table 1. Data and error components for the example in Figure 1. For the squared errors and their components, the means of the rows form MSE, $\mathrm{MSE_s}$, and MSE_u. For the absolute errors, the mean of the $|P_i - O_i|$ row also forms MAE, but the other three rows that have absolute values are used as weights (b, p_i, and u_i) to determine MAE_b, MAE_p and MAE_u (see Eqs. 14–16).

Component	1	2	3	4	5	6	7
Observed (O_i)	-4.0	-2.0	1.0	2.0	3.0	4.0	5.0
Predicted (P_i)	0.5	-4.0	0.0	1.5	1.0	4.0	1.0
$(P_i - O_i)^2$	20.2500	4.0000	1.0000	0.2500	4.0000	0	16.0000
$(\hat{P}_i - O_i)^2$	5.0625	1.2732	0.3070	1.2429	2.8079	5.0019	7.8249
$(P_i - \hat{P}_i)^2$	5.0625	9.7868	0.1989	0.3781	0.1052	5.0019	1.4465
$\lvert P_i - O_i \rvert$	4.5000	2.0000	1.0000	0.5000	2.0000	0	4.0000
$b = \lvert \mathrm{MBE} \rvert$	0.7143	0.7143	0.7143	0.7143	0.7143	0.7143	0.7143
$p_i = \lvert \hat{P}'_i - O_i \rvert$	2.9643	1.8427	0.1602	0.4006	0.9614	1.5222	2.0830
$u_i = \lvert P'_i - \hat{P}'_i \rvert$	2.2500	3.1284	0.4459	0.6149	0.3243	2.2365	1.2027

Unsystematic Error

After accounting for bias and proportionality errors, the remaining error is related to scatter about $\hat{P}'$. Analogous to the way that the individual components of MSE_u are formed, weights for the relative importance of each prediction's unsystematic error are determined using the difference between the unbiased predictions and the unbiased regression values:

$$u_i = |P'_i - \hat{P}'_i| \ . \tag{12}$$

Once again, if OLS regression is used for $\hat{P}'_i$, then the biased predictions and regression values produce the same weights:

$$u_i = |P'_i - \hat{P}'_i| = |P_i - \hat{P}_i| \ . \tag{13}$$

Three-Component Decomposition of MAE

The three weights for bias, proportionality, and unsystematic error developed above now can be used to scale the individual components of absolute error:

$$\mathrm{MAE}_b = \sum_{i=1}^{n} \frac{b}{b + p_i + u_i} |P_i - O_i| \ , \tag{14}$$

$$\mathrm{MAE}_p = \sum_{i=1}^{n} \frac{p_i}{b + p_i + u_i} |P_i - O_i| \ , \tag{15}$$

$$\mathrm{MAE}_u = \sum_{i=1}^{n} \frac{u_i}{b + p_i + u_i} |P_i - O_i| \ . \tag{16}$$

A clear advantage of this weight-based decomposition of average error is that it uses MAE rather than MSE as the baseline. Another advantage is that predictions that have no error do not contribute to the components. This was not the case with the MSE-based decomposition, where predictions that have no error can substantially influence the values of MSE_s and MSE_u (e.g., the point that lies directly on the 1:1 line

in the upper right panel of Fig. 1 contributes substantially to both $\mathrm{MSE_s}$ and $\mathrm{MSE_u}$ despite being error-free).

It is possible for the denominator within these summations (i.e., $b + s_i + u_i$) to be zero, but that can only occur when a model has no bias and the regression line passes through a predicted value that has no error (i.e., when $b = 0$ and $P_i = \hat{P}_i = O_i$). If that rare model-prediction event occurs, those elements with $b + p_i + u_i = 0$ can simply be excluded from the summation.

Using these definitions, MAE_b, MAE_p, and MAE_u are conservative and must sum to MAE:

$$\mathrm{MAE} = \mathrm{MAE}_b + \mathrm{MAE}_p + \mathrm{MAE}_u \, . \tag{17}$$

As with MSE_s and MSE_u, it is instructive to form ratios (i.e., $\mathrm{MAE}_b/\mathrm{MAE}$, $\mathrm{MAE}_p/\mathrm{MAE}$, and $\mathrm{MAE}_u/\mathrm{MAE}$) to identify the proportion of total error contributed by each component.

An Example of Model-Estimation Errors

To illustrate the properties of our newly derived measures of model error, we use a tree-ring based reconstruction model developed by [14]. This reconstruction provides over 1200 years of annually resolved flow predictions for the Upper Colorado River at Lee's Ferry. The large majority of the annual flow in the Colorado River comes from upstream of Lee's Ferry [15], so the reconstruction is an essential indicator of historical water availability for the Colorado River. The observed and reconstructed flow are water-year totals for a large river and, therefore, are reported in billions of cubic meters per year. Both the observed data and reconstructed values are based on estimates of "naturalized" streamflow, which corrects for the anthropogenic alterations of flow (i.e., reservoirs, irrigation, etc.). In a recent article [13], the model was bias-corrected so that its empirical probability distribution better matched that of the observations (e.g., compare Fig. 2a and 2b). Here, we employ our new decomposition of model errors to compare the different sources of error in the original and bias-corrected reconstruction.

Fig 2. Model-estimation errors before and after bias correction. Model-estimation errors for (a) the reconstruction of Upper Colorado River flow from [14] and (b) the same reconstruction after applying the bias-correction procedure of [13].

Prior to bias correction (Fig. 2a), the Upper Colorado River reconstruction has low overall error, with a MAE of 2.12 billion m^3 (i.e., when compared to the $\bar{O}$ of 18.53 billion m^3). The small value of $\mathrm{MAE_b}$ (0.08 billion m^3) also shows that the reconstruction model faithfully reproduces the observed mean. From the scatterplot and the substantial amount (34%) of error in $\mathrm{MAE_p}$, however, it is clear that high flow years are underestimated (and, to a lesser extent, low flows are overestimated). Even with these substantial proportionality errors, the majority (62%) of the mean absolute error is in $\mathrm{MAE_u}$), which is desirable (i.e., the majority of error is random or unsystematic). At the same time, the traditional decomposition into $\mathrm{MSE_s}$ and $\mathrm{MSE_u}$ masks the distinction between bias and proportionality error while also providing an underestimate of these combined systematic errors because it is inflating the "average" unsystematic error ($\mathrm{MSE_u}$) by squaring the model-predicted deviations from the regression line. As a result, the MSE-based measures suggest that there is little room for improvement when there is.

Bias correction (Fig. 2b) produces a reconstruction model that has similar MAE (2.05 billion m^3) to the original model. But, bias correction has produced much lower error in the two systematic terms of MAE, reducing $\mathrm{MAE_b}$ to 3% and $\mathrm{MAE_p}$ to 19% of

MAE. From the slope of the regression line, however, it is clear that there still is some proportionality error that the bias-correction procedure has not entirely removed. The MSE-based measures present a rosier picture of the reduction of systematic error, again due to the inflation of the unsystematic error produced by the squaring of the deviations around the regression line. Overall, the MAE-based approach shows that there is still room for additional improvement in the original reconstruction (Fig. 2a) and in the bias correction procedure (Fig. 2b) than is evident in the MSE-based measures. In particular, the additional systematic component introduced here, $\mathrm{MAE_p}$, suggests that high flows still need to be adjusted upward.

Conclusions

Traditional decomposition of sums-of-squared errors into systematic ($\mathrm{MSE_s}$) and unsystematic ($\mathrm{MSE_u}$) components has been a po[illegible]ful approach for characterizing the different components of model error. These sums-of-squares-based measures, however, have been shown to be imprecise and, at times, deceptive indicators of average error and its constituents. As a result, evaluations of model estimates and predictions should increasingly use absolute-value-based error measures such as MAE. To fill the need for a decomposition of MAE into its constituent components, we present new measures that are formed as weighted averages of the overall error. As a result, MAE can now be decomposed into three components that represent bias ($\mathrm{MAE_b}$), proportionality ($\mathrm{MAE_p}$), and unsystematic ($\mathrm{MSE_u}$) errors. These measures provide a more intrepretable standard for evaluating model errors while also pointing to more specific types of error that may be reduced.

References

1. Willmott CJ, Robeson SM, Matsuura K. Climate and other models may be more accurate than reported. EOS. 2017 98.

2. Willmott CJ, Robeson SM, Matsuura K., Ficklin DL Assessment of three dimensionless measures of model performance. Environ Mod Softw. 2015 73: 167–174.

3. Nash JE, Sutcliffe JV River flow forecasting through conceptual models part I—A discussion of principles. J Hydrol. 1970 10: 282–290.

4. Legates DR, McCabe Jr, GJ Evaluating the use of “goodness-of-fit” measures in hydrologic and hydroclimatic model validation. Wat Resour Res. 1999 35: 233–241.

5. Willmott CJ, Robeson SM, Matsuura K A refined index of model performance. Intl J Climatol. 2012 32: 2088–2094.

6. Jackson EK, Roberts W, Nelsen B, Williams GP, Nelson EJ, Ames Dp Introductory overview: Error metrics for hydrologic modelling–A review of common practices and an open source library to facilitate use and adoption. Environ Mod Softw. 2019 119: 32–48.

7. Willmott CJ, Matsuura K Advantages of the mean absolute error (MAE) over the root mean square error (RMSE) in assessing average model performance. Clim Res. 2005 30: 79–82.

8. Gao, J Bias-variance decomposition of absolute errors for diagnosing regression models of continuous data. Patterns. 2: 100309.
9. Mielke PW, Berry K Permutation methods: a distance function approach, Springer; 2007.
10. Pontius RG, Thontteh O, Chen H Components of information for multiple resolution comparison between maps that share a real variable. Environ Ecol Stat. 15: 111–142.
11. Willmott CJ, Matsuura K, Robeson SM. Ambiguities inherent in sums-of-squares-based error statistics. Atmos Environ. 2009 43: 749–752.
12. Willmott CJ On the validation of models Phys Geog. 1981 2(2):184-94.
13. Robeson SM, Maxwell JT, Ficklin, DL Bias correction of paleoclimatic reconstructions: A new look at 1,200+ years of Upper Colorado River flow. Geophys Res Lett. 2020 e2019GL086689.
14. Meko DM, Woodhouse CA, Baisan CA, Knight T, Lukas JJ, Hughes MK, Salzer MW Medieval drought in the upper Colorado River Basin. Geophys Res Lett. 2007 L10705, doi:10.1029/2007GL029988.
15. Christensen NS, Lettenmaier DP A multimodel ensemble approach to assessment of climate change impacts on the hydrology and water resources of the Colorado River Basin, Hydrol Earth Sys Sci. 2007 11: 1417–1434.

Figure 1

Click here to access/download;Figure;Fig1.tif

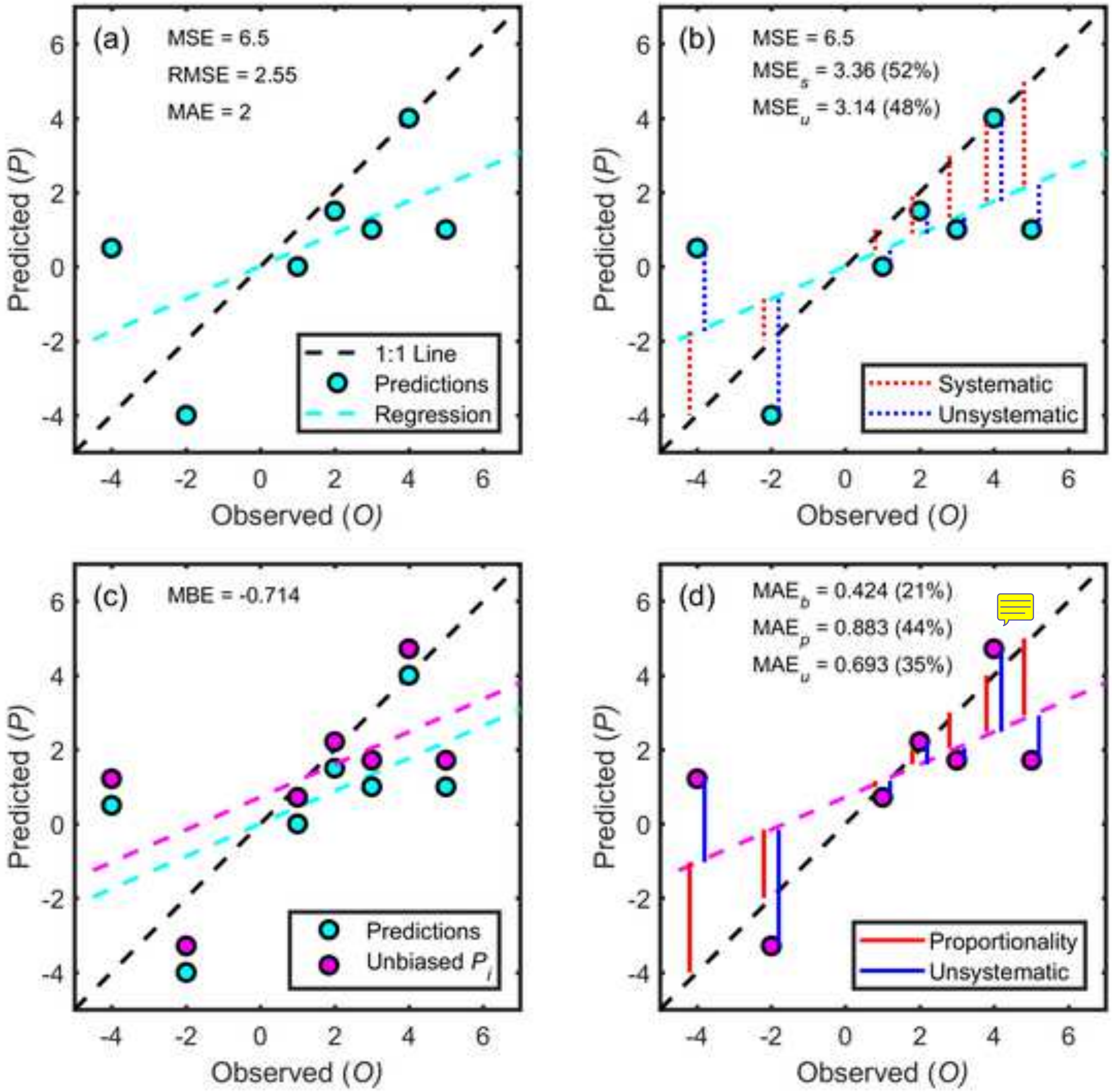

Figure 2

Click here to access/download;Figure;Fig2.tif

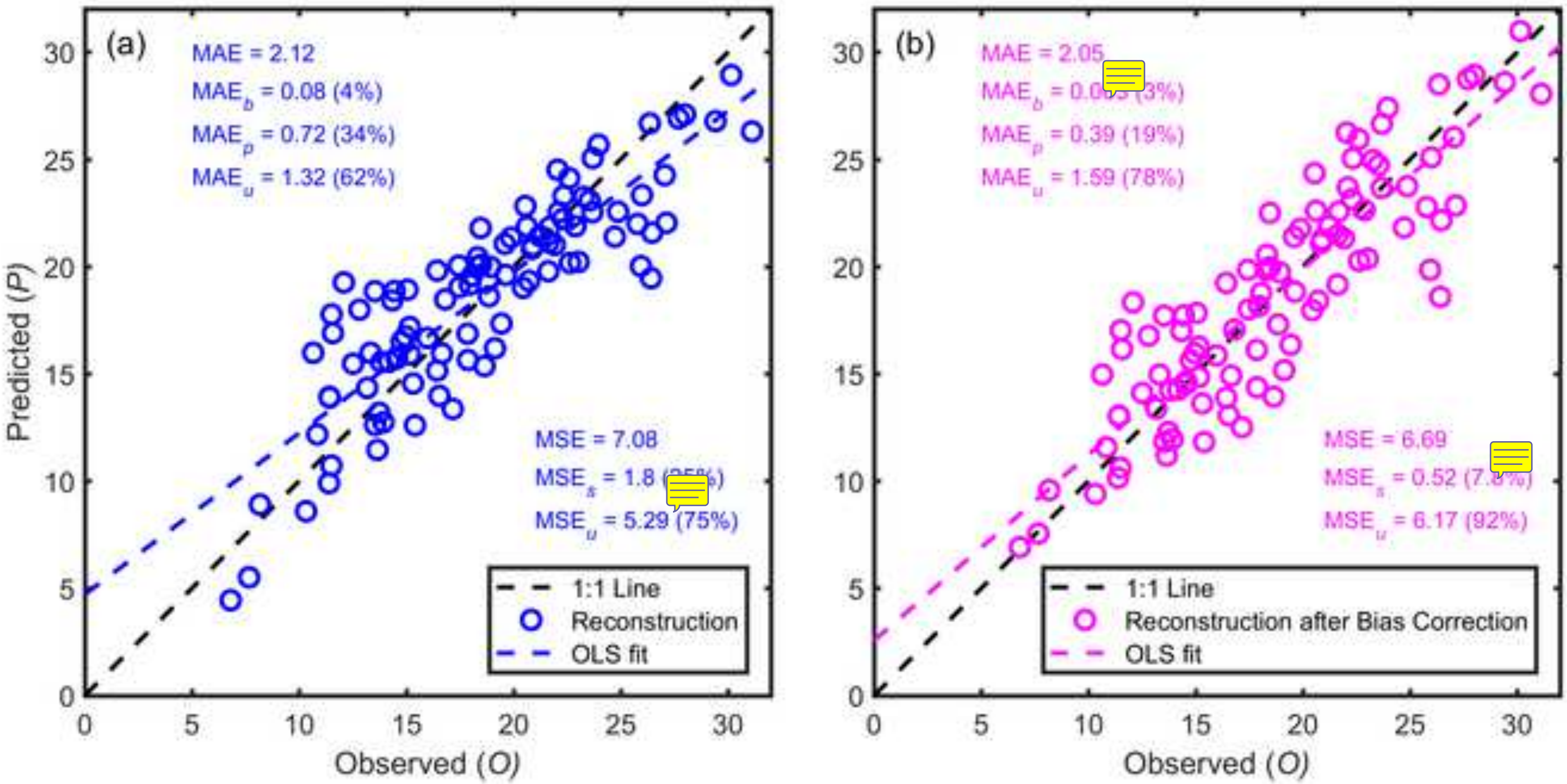

Supplement: Attachment — Submitted filename: PONE-D-22-17609_reviewer.pdf [file pone.0279774.s002.pdf]
