# Supplementary material for: Decomposition of the mean absolute error (MAE) into systematic and unsystematic components
Source: PLoS One. 2023 Feb 17;18(2):e0279774. doi: 10.1371/journal.pone.0279774 (PMC9937461; doi:10.1371/journal.pone.0279774)
Supplement: Attachment — Submitted filename: Decomposition_MAE_Response_to_Review.pdf [file pone.0279774.s003.pdf]

Decomposition of the mean absolute error (MAE) into systematic and unsystematic components

Response to Reviews

(author responses in blue text below)

Reviewer #1

My areas of expertise focus on metrics to measure error and change, which is the exact topic of the submitted manuscript. I have thought about the concepts of the submitted manuscript for decades. Therefore, I report with confidence that the submitted manuscript is a brilliant major breakthrough. I inserted several comments in the PDF as I read. I ask the authors to consider those comments to increase clarity of the manuscript. The submitted manuscript is short, which is a strength. The manuscript states only what is necessary, which any scientific manuscript should do. The manuscript proposes a method to fix the flawed popular paradigm of squared deviations. The authors' new method makes much better sense than the popular paradigm. Previous methods that have used Mean Absolute Error (Pontius Jr 2022) do not separate the Mean Absolute Error into as many helpful components as the proposed manuscript does. The manuscript illustrates how the new method has helpful practical implications. Below are ideas to make the manuscript even stronger than it already is.

Thank you so much for your comments and the very useful feedback throughout your review.

My browser could not activate the link where the authors have posted the data at https://www.ncdc.noaa.gov/dataaccess/paleoclimatology%E2%80%90data

Our apologies. We have corrected the link, which now goes directly to the NOAA site for the particular data used. It also is given below:
https://www.ncei.noaa.gov/access/paleo-search/study/28810

The example is helpful. It would be more helpful to have a column at the right in Table 1 to show the sum. It would be nicer to have the numbers be simpler such as all whole numbers for Pi and Oi, and to have n be a number that makes easier division than by 7.

We modified our example to use a set of 6 numbers. We also added the summation column at the far right. The figure has been updated accordingly.

In figure 1d, it is not immediately clear to me why MAEb = 0.424 rather than |-0.714|, which is the absolute bias in figure 1c. The reader would understand better if the revised manuscript were to have a sentence to explain why |Bias| does not equal MAEb.

We have added the following explanation at the end of the section that discusses Fig. 1, as well as the additional recommendation to continue examining MBE:

The constraints within the weighted decomposition of MAE diminish MAE_b relative to the magnitude of MBE. MBE, therefore, remains a useful metric to be reported when analyzing model error.

Figures 1 and 2 should be consistent in the number of digits in the results. Report the % to the nearest whole number. The other numbers should have exactly two decimal places.

We made these corrections.

I thank the authors for using sequential line numbers.
In line 108, I think it would be clearer to eliminate “are conservative and must”
In line 137, the meaning of the quotes around average is unclear. I find the language is imprecise when I see quotes like that.
In line 155, replace “powerful” with “popular”.

We made minor edits to address all of these comments.

In lines 139-140, the implication is profound. Congratulations on the creation of a helpful method.

Thank you!

Readers will be eager to have computer code, say in R, which would inspire more rapid adoption by researchers.

We now provide R and Matlab functions for these calculations in the Supporting Information.

I hope the authors find this review helpful, as I intend it to be. The authors have achieved a major accomplishment.

We found this review extremely helpful and appreciate your positive assessment.

LITERATURE
Pontius Jr, Robert Gilmore. 2022. Metrics That Make a Difference: How to Analyze Change and Error. Advances in Geographic Information Science. Cham: Springer International Publishing. https://doi.org/10.1007/978-3-030-70765-1.

This reference was added and we also made the suggested editorial changes that were in the annotated PDF.

Reviewer #2

In the present paper, the authors propose the decomposition of the Mean Absolute Error into three components which represent the model bias, proportionality, and unsystematic components. The error components are clearly explained through a synthetic example and a data sample. Therefore, the present study improves the standard measures for evaluating model errors significantly. The analysis of the three components makes it possible to understand their different contribution. Overall, the paper is well written and well organized, hence the decision is to accept after minor revisions.

Thank you very much for your comments and for the overall positive assessment of our work.

Points of attention:

• The data link is not working: https://www.ncdc.noaa.gov/dataaccess/ paleoclimatology-data

Our apologies. We have added the corrected link, which now goes directly to the NOAA site for the particular data used. It also is given below:
https://www.ncei.noaa.gov/access/paleo-search/study/28810

• Page 2 line 16. remove parenthesis
• Page 2 line 41. remove parenthesis

We made these two corrections.

• Page 4 equation (14-16). Should be divided by n.

Thank you for catching this error. It has been corrected.

• Page 5 equation (17) holds due to the definitions in (14)-(16).

We changed the text here slightly to clarify this point.
